# Law

**Published:** 1884-11

**Authors:** 


### EDITORIAL.

### Law.

Below we give an amendment to the law regulating the practice of dentistry in the State of New Jersey, passed last March. This is a very important movement, as will be seen by reference to the amendment, that, hereafter, in the State of New Jersey, no one can enter the practice of dentistry unless he has a diploma from the faculty of a reputable dental college. This is undoubtedly a movement in the right direction. Whether every State is prepared to take such a position may be a question in the minds of some; however, we can not see why any other State may not take this step as well as New Jersey. The law also has a registration clause, which is a very important matter, and the law of every State should have a similar provision. Movements such as are here enacted, speak great things for the profession in the future. This places the responsibility for the character of the dental profession chiefly in the hands of the colleges; they should therefore assume such a position as will answer the demand in this direction. They should also, each and every one, occupy such a position that no question could arise as to their standing or efficiency. No college can afford to have the question of its reputation arise in the minds of any.

The progress that has been made in the matter of regulating the practice of dentistry, within the last fifteen years, is truly remarkable. About that time, the first laws for this purpose were enacted—they were then regarded by the most sanguine as experimental—much doubt was entertained as to their efficiency, or, indeed, as to their being of any service, while a large proportion of the profession, and a great many of the better class, too, regarded such enactments as altogether impracticable and unnecessary, and some laughed the matter to scorn. Notwithstanding this, however, these enactments have proved of inestimable value--influencing the profession to a better state and a higher standard.

More than one-half of the States of the Union have such laws, and all the others desire to have them, and most of the States in which the laws exist, desire to have them improved, and doubtless they should be. It is a matter, then, that each State Dental Society should consider; and take such action as would secure the most efficient enactments, and it would seem desirable that there be uniformity, so near as possible, in this. There is no reason, except prejudice and fear, why the laws of all the States might not have the same requirements as are set forth in this amendment; namely, the endorsement of the faculty of a reputable college, and a proper registration, as a requisite for entering upon the practice of dentistry. This, while it might not only prevent quackery and incompetency, would certainly very much lessen both.

"A SUPPLEMENT to an act entitled 'An act to regulate the practice of dentistry, and to protect the people against empiricism in relation thereto in the State of New Jersey,' approved March fourteenth, one thousand eight hundred and seventy-three.

"1. BE IT ENACTED *by the Senate and General Assembly of the State of New Jersey*, That the first section of the act to which this is a supplement shall be amended so as to read as follows:

["1. BE IT ENACTED *by the Senate and General Assembly of the State of New Jersey*, That from and after the passage of this act it shall be unlawful for any person, not now lawfully practicing, to engage in the practice of dentistry in the State of New Jersey, unless said person has graduated and received a diploma from the faculty of a reputable dental college, chartered under the authority of some one of the United States, and that any person hereafter engaging in the practice of dentistry in the State, shall, within one month after commencing such practice register his name in a book, kept for that purpose in the County Clerk's office of said county in which he shall have engaged in the practice of dentistry, giving his name and the name of the dental college of which he is a graduate, and the name of the place in which he shall have engaged in practice, and for which registry the County Clerk shall be entitled to demand and receive from each person registering the sum of fifty cents, and any person violating any of the provisions of this act shall be liable to the penalties prescribed in the sixth section of the act to which this is a supplement.]

"Approved March 27th, 1884."

